# Tumor necrosis factor alpha maintains denervation-induced homeostatic synaptic plasticity of mouse dentate granule cells

**DOI:** 10.3389/fncel.2013.00257

**Published:** 2013-12-18

**Authors:** Denise Becker, Nadine Zahn, Thomas Deller, Andreas Vlachos

**Affiliations:** Institute of Clinical Neuroanatomy, Neuroscience Center, Goethe-University FrankfurtFrankfurt, Germany

**Keywords:** entorhinal cortex lesion, homeostatic synaptic scaling, astrocytes, brain injury, organotypic slice cultures

## Abstract

Neurons which lose part of their input respond with a compensatory increase in excitatory synaptic strength. This observation is of particular interest in the context of neurological diseases, which are accompanied by the loss of neurons and subsequent denervation of connected brain regions. However, while the cellular and molecular mechanisms of pharmacologically induced homeostatic synaptic plasticity have been identified to a certain degree, denervation-induced homeostatic synaptic plasticity remains not well understood. Here, we employed the entorhinal denervation *in vitro* model to study the role of tumor necrosis factor alpha (TNFα) on changes in excitatory synaptic strength of mouse dentate granule cells following partial deafferentation. Our experiments disclose that TNFα is required for the maintenance of a compensatory increase in excitatory synaptic strength at 3–4 days post lesion (dpl), but not for the induction of synaptic scaling at 1–2 dpl. Furthermore, laser capture microdissection combined with quantitative PCR demonstrates an increase in TNFα-mRNA levels in the denervated zone, which is consistent with our previous finding on a local, i.e., layer-specific increase in excitatory synaptic strength at 3–4 dpl. Immunostainings for the glial fibrillary acidic protein and TNFα suggest that astrocytes are a source of TNFα in our experimental setting. We conclude that TNFα-signaling is a major regulatory system that aims at *maintaining* the homeostatic synaptic response of denervated neurons.

## INTRODUCTION

Homeostatic synaptic plasticity is a slow adaptive mechanism which allows neurons to adjust their synaptic strength to perturbations in network activity. It aims at keeping the firing rate of neurons within a dynamic range and is considered fundamental for the normal functioning of the central nervous system. Hence, in response to a prolonged reduction in network activity neurons increase (“scale up”) the strength of their excitatory synapses (Turrigiano, 2012; Vitureira et al., 2012; Wang et al., 2012; Davis, 2013). Using entorhinal denervation *in vitro* we recently showed that homeostatic synaptic strengthening of excitatory synapses is observed in denervated neuronal networks (Vlachos et al., 2012a, 2013a,b). This observation indicates that homeostatic synaptic responses could play an important role in a broad range of neurological diseases, which are accompanied by the loss of central neurons and subsequent denervation of connected brain regions. However, the molecular pathways involved in the regulation of denervation-induced homeostatic synaptic plasticity remain incompletely understood.

One of the factors suggested to control homeostatic synaptic scaling following prolonged blockade of sodium channels with tetrodotoxin (TTX) or pharmacological inhibition of ionotropic glutamate receptors, is the pro-inflammatory cytokine TNFα (Stellwagen and Malenka, 2006). While it has been shown that TNFα affects synaptic strength (Beattie et al., 2002; Stellwagen et al., 2005; Leonoudakis et al., 2008; Santello et al., 2011; He et al., 2012; Pribiag and Stellwagen, 2013), its precise role in synaptic plasticity remains controversial. Recently experimental evidence has been provided that TNFα may act as a permissive rather than instructive factor (Steinmetz and Turrigiano, 2010). Likewise, its impact on synaptic plasticity under pathological conditions remains not well understood (for a recent review on the role of TNFα in synaptic plasticity see Santello and Volterra, 2012).

Here, we studied the role of TNFα in denervation-induced synaptic plasticity using mature (≥18 days *in vitro*; div) entorhino-hippocampal slice cultures (Del Turco and Deller, 2007). In these cultures the axonal projection from the entorhinal cortex (EC) to the outer molecular layer (OML) can be transected by removing the EC from the culturing dish (e.g., Vlachos et al., 2013a). This leads to the partial deafferentation of dentate granule cells in the OML, without directly damaging the dentate gyrus (DG; Müller et al., 2010). Using pharmacological and genetic approaches we provide experimental evidence that denervation-induced synaptic plasticity is divided into a TNFα-independent early phase [1–2 days postlesion (dpl)] and a TNFα-dependent late phase (3–4 dpl). Astrocytes seem to be a major source of TNFα in our experimental setting. These results suggest an important role for TNFα in maintaining synaptic scaling responses in denervated neuronal networks, which could be of relevance in the context of neurological diseases in which neuronal death and denervation occur.

## MATERIALS AND METHODS

### PREPARATION OF SLICE CULTURES

Experimental procedures were performed in agreement with the German law on the use of laboratory animals and approved by the animal welfare officer of Goethe-University Frankfurt (Faculty of Medicine). Entorhino-hippocampal slice cultures were prepared at postnatal day 4–5 as previously described (e.g., Becker et al., 2012; Vlachos et al., 2013a). C57BL/6J and TNFα-deficient mice (and their wildtype littermates) of either sex were used (Pasparakis et al., 1996; Golan et al., 2004; obtained from Jackson Laboratories, USA). Slice cultures were allowed to mature for ≥18 div in humidified atmosphere with 5% CO_2_ at 35°C before experimental assessment.

### ENTORHINAL CORTEX LESION

**FIGURE 1:**
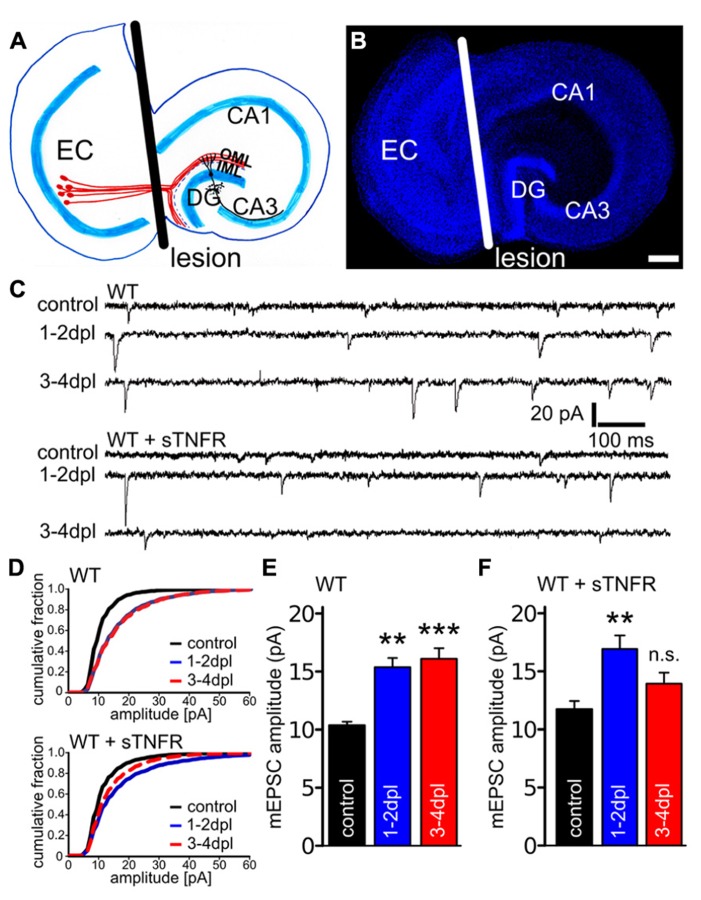
**Denervation-induced homeostatic synaptic strengthening is impaired at 3–4 dpl in the presence of soluble TNF-receptor.** **(A)** Schematic of an organotypic entorhino-hippocampal slice culture. Neurons in the entorhinal cortex (EC, red) project their axons into the outer molecular layer (OML) of the dentate gyrus (DG). By cutting through these axons and by removing the EC from the culture dish (plane of transection, black line), distal dendrites of dentate granule cells are denervated. IML, inner molecular layer. **(B)** Example of an entorhino-hippocampal slice culture stained with ToPRO nuclear stain (blue). The white line depicts the plane of transection. Scale bar: 200 μm. **(C)** Sample traces of mEPSC recordings from granule cells of non-denervated control and denervated wildtype slice cultures untreated and treated with soluble TNF-receptor (sTNFR). **(D–F)** While a compensatory increase in mEPSC amplitudes was observed in denervated wildtype cultures (both at 1–2 dpl and 3–4 dpl; *n* = 10–12 neurons per group, from four to five cultures each), granule cells treated with sTNFR were impaired in their ability to maintain increased mEPSC amplitudes at 3–4 dpl (*n* = 9–11 neurons per group, from three to four cultures each). Data represent mean ± SEM; **p < 0.01; ***p < 0.001; n.s., not significant.

Slice cultures (18–25 div) were transected using a sterile scalpel blade (**Figures 1A,B**; e.g., Vlachos et al., 2012a, 2013a). To ensure complete and permanent separation of the EC from the hippocampus, the EC was removed from the culturing dish.

### PERFORANT PATH TRACING

**FIGURE 2:**
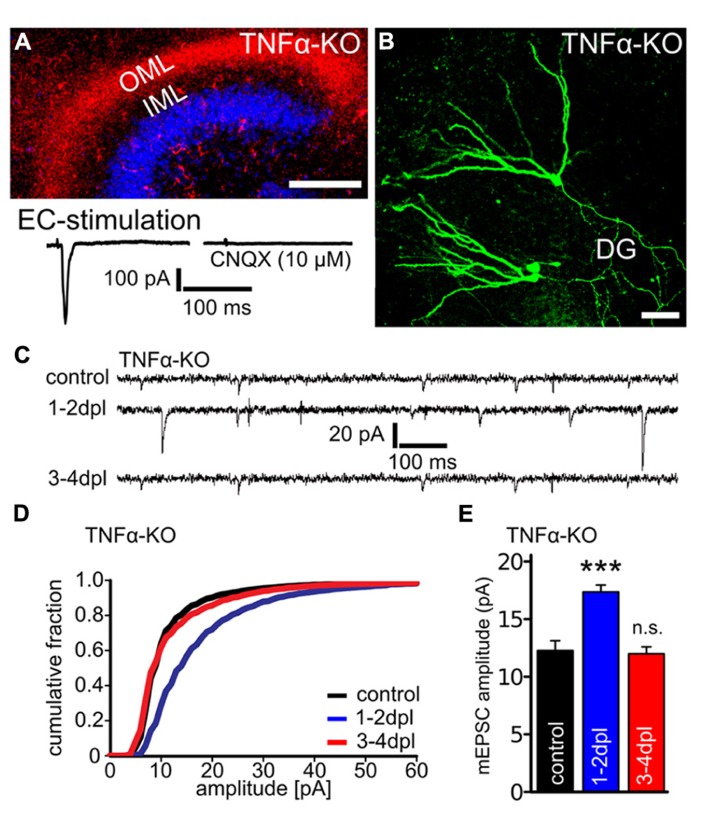
**Denervation-induced homeostatic synaptic strengthening is not observed in granule cells of TNFα-deficient slice cultures at 3–4 dpl.** **(A)** Mini-Ruby tracing of entorhino-hippocampal axons (red; ToPRO nuclear staining, blue) and electrical stimulations of the entorhinal cortex (EC) while recording evoked EPSCs from dentate granule cells revealed an intact and functional entorhino-hippocampal projection in slice cultures prepared from TNFα-deficient mice (TNFα-KO; three independent experiments each, up to 50 traces averaged per neuron). Evoked EPSCs (amplitude: 369 ± 102 pA) could be blocked by the AMPA-receptor antagonist CNQX (10 μM; amplitude: 9.6 ± 2.1 pA). Scale bar: 200 μm. **(B)** Patched granule cells were filled with biocytin and *post hoc* identified using Alexa568- or Alexa488-streptavidin. Scale bar: 50 μm. **(C–E)** Whole-cell patch-clamp recordings from granule cells of TNFα-deficient slice cultures revealed an increase in the mEPSC amplitudes at 1–2 dpl but not at 3–4 dpl (*n* = 12–16 neurons per group, from six to eight cultures each). Data represent mean ± SEM; ***p < 0.001; n.s., not significant.

Anterograde tracing of the entorhino-hippocampal pathway with biotinylated and rhodamine conjugated dextranamine Mini-Ruby (**Figure 2A**; Molecular Probes, USA) was performed as described previously (Kluge et al., 1998; Prang et al., 2003; Vlachos et al., 2012a).

### WHOLE-CELL PATCH-CLAMP RECORDINGS

Whole-cell voltage-clamp recordings and *post hoc* identification of recorded neurons were carried out as previously described (Vlachos et al., 2012a). Age- and time-matched non-denervated cultures prepared from the same animal or littermate animals served as controls. Non-denervated control (or untreated) cultures were recorded alternating with the recordings of denervated and/or treated cultures (c.f., Vlachos et al., 2012a). All recordings were performed at 35°C in artificial cerebrospinal fluid (ACSF; 126 mM NaCl, 2.5 mM KCl, 26 mM NaHCO_3_, 1.25 mM NaH_2_PO_4_, 2 mM CaCl_2_, 2 mM MgCl_2_, and 10 mM glucose) saturated with 95% O_2_/5% CO_2__._ For miniature excitatory postsynaptic current (mEPSC) – recordings 10 μM D-APV, 10 μM SR-95531 and 0.5 μM TTX were added to ACSF. Patch-pipettes contained 126 mM K-gluconate, 4 mM KCl, 4 mM ATP-Mg, 0.3 mM GTP-Na_2_, 10 mM PO-Creatine, 10 mM HEPES and 0.3% Biocytin (pH = 7.25 with KOH, 290 mOsm with sucrose). Recordings were carried out at a holding potential of -70 mV. Series resistance was monitored in 2 min intervals, and recordings were discarded if the series resistance and leak current changed significantly and/or reached ≥30 MΩ or ≥50 pA, respectively.

### LOCAL ELECTRICAL STIMULATION

A bipolar stimulation electrode (NE-200, 0.5 mm tip separation, Rhodes Medical Instruments, USA) was placed on the EC and current square-wave pulses (50 μA; 1 ms at 10 Hz) were generated by a stimulus generator (STG1002 Multichannel Systems, Germany) while recording evoked excitatory postsynaptic currents from individual granule cells (**Figure 2A**; c.f. Vlachos et al., 2012a).

### DRUG TREATMENTS

Denervated and non-denervated slice cultures were treated with recombinant TNFα (0.1 μg/ml; Sigma, Germany) for 1 or 2 days. Soluble recombinant TNF-receptor 1 (sTNFR, 10 μg/ml, Catalog number: 425-R1-050, R&D systems, USA) was applied directly after the lesion for up to 4 days (incubation medium replaced once with fresh sTNFR-containing medium at 2 days).

### LASER CAPTURE MICRODISSECTION OF RE-SLICED CULTURES

Slice cultures were washed with phosphate buffered saline (PBS; 0.1 M, pH 7.4), shock frozen at -80°C in tissue freezing medium (Leica Microsystems, Germany), re-sliced into 10 μm thick slices on a cryostat (Leica CM 3050 S) and mounted on PET foil metal frames (Leica, Germany) as described previously (Vlachos et al., 2013b). Re-sliced cultures were fixed in ice-cold acetone for 1 min and incubated with 0.1% toluidine blue (Merck, Germany) at room temperature for 1 min, before rinsing in ultrapure water (DNase/RNase free, Invitrogen, USA) and 70% ethanol. PET foil metal frames were mounted on a Leica DM 6000B laser capture microdissection (LMD) system (Leica Microsystems, Germany) with the section facing downward (Burbach et al., 2003). After adjusting intensity, aperture, and cutting velocity, the pulsed ultraviolet laser beam was carefully directed along the borders of the respective hippocampal layers of interest using a 20× objective lens (Leica Laser Microdissection, Software Version 7.4.1.4853). Tissue from the OML, the inner molecular layer (IML) and the granule cell layer (GCL) of the suprapyramidal blade of the DG were collected. Microdissected tissue was transferred by gravity into microcentrifuge tube caps placed underneath the sections, filled with 50 μl guanidine isothiocyanate (GITC)-containing buffer (RLT Buffer, RNeasy Mini Kit, Qiagen, Germany) with 1% β-mercaptoethanol (AppliChem GmbH; Germany). Successful tissue collection was verified by visually inspecting the content of the tube caps. All samples were frozen and stored at -80°C.

### ISOLATING RNA AND qPCR

RNA was isolated using the RNeasy^®^ MicroPlus Kit (Qiagen, Germany). Purified RNA was transcribed into cDNA with the High Capacity cDNA Reverse Transcription Kit (Applied Biosystems, USA). All kits and assays were used according to the manufacturer’s instructions. The cDNA was amplified using the TaqMan^®^ PreAmp Master Mix Kit (Applied Biosystems, USA) using 5 μl PreAmp Master Mix (Applied Biosystems, USA) + 2.5 μl cDNA + 2.5 μl Assay Mix [TaqMan Gene Expression(TM)-Assay (GAPDH: 4352932E; TNFα: Mm00443258_m1) from Applied Biosystems, USA] with a standard amplification protocol (14 cycles: 95°C for 15 s; 60°C for 4 min). Amplified cDNAs were diluted 1:20 in ultrapure water and subjected to quantitative PCR (qPCR; StepOnePlus, Applied Biosystems, USA) using a standard amplification program (1 cycle of 50°C for 2 min, 1 cycle of 95°C for 10 min, 40 cycles of 95°C for 15 s and 60°C for 60 s; cut off at 36 cycles; average *C_T_*-value was: 22.7 ± 0.7 cycles).

### FLUORESCENCE *IN SITU* HYBRIDIZATION

*In situ* hybridization was performed using a biotin-labeled oligo-DNA probe (5′ CT TCT CAT CCC TTT GGG GAC CGA TCA CC 3′) directed against murine TNFα-mRNA (Fenger et al., 2006; Lambertsen et al., 2007). All steps were carried out under RNase-free conditions. Buffers and solutions were prepared with DEPC-treated water. Wildtype cultures (2 dpl) were fixed for 30 min in 4% (*w/v*) paraformaldehyde (PFA) containing PBS followed by 2% (*w/v*) PFA and 30% (*w/v*) sucrose in PBS at 4°C overnight. Fixed cultures were snap-frozen in tissue freezing medium (Leica) on dry-ice and re-sliced into 18 μm sections on a cryostat (Leica CM 3050 S). Sections were mounted on “Superfrost plus”-microscope slides (Thermo Scientific, USA), quenched in 0.3% (*v/v*) H_2_O_2_ and washed twice with PBS prior to Avidin/Biotin Blocking (Invitrogen, USA). The oligo-DNA probe (200 ng/ml) was added to the hybridization buffer [4 × SSC, 50% (*v/v*) Formamide, 200 mg/ml dextran sulfate sodium salt, 0.25 mg/ml ssDNA, 0.25 mg/ml tRNA, 0.01 M DTT and 1× Denhardt’s solution; at 37°C] and sections were incubated with the buffer in a humidified chamber at 37°C overnight. Thereafter sections were washed twice for 15 min at 55°C in 1 × SSC and 0.5 × SSC [both containing 50% (*v/v*) Formamide and 0.1% (*v/v*) Tween-20] and the formamide was removed by washing in Maleic acid buffer [100 mM Maleic acid, 150 mM NaCl, 0.1% (*v/v*) Tween-20, pH 7.5]. The TSA^TM^-biotin system (PerkinElmer, USA) was used for signal amplification and deposited biotin was visualized by incubating the sections in Alexa Fluor 568 conjugated Streptavidin [1:1000 in TNB (buffer containing 0.1 M TRIS-HCl, 0.15 M NaCl and 1% (*v/v*) provided blocking reagent) for 30 min; Molecular Probes, USA].

Counterstaining for the glial fibrillary acidic protein (GFAP) was performed using a monoclonal anti-GFAP antibody (1:100 in TNB for 10 min; Sigma-Aldrich, Germany) followed by Alexa Fluor 488 conjugated mouse secondary antibody (1:50 in TNB for 10 min, Molecular Probes, USA). Sections were washed three times in PBS and mounted on microscope slides using fluorescence mounting medium (Dako, Denmark).

### IMMUNOHISTOCHEMISTRY

Cultures were fixed in a solution of 4% (*w/v*) PFA and 4% (*w/v*) sucrose in PBS for 1 h, followed by 1 h in 2% (*w/v*) PFA and 30% (*w/v*) sucrose in PBS. Fixed slice cultures were thoroughly washed, resliced into 30 μm sections (Leica VT 1000S, Germany), and stained with antibodies against TNFα (1:500; Abcam, AB66579) and GFAP (1:2000; Sigma-Aldrich, Germany) in PBS with 10% (*v/v*) normal horse serum and 0.1% (*v/v*) Triton X-100 based on a previously described protocol (Vlachos et al., 2013a). Secondary antibodies (goat anti-rabbit Alexa568 and goat anti-mouse Alexa488; Invitrogen, USA) were used at 1:2000 in PBS with 10% (*v/v*) normal horse serum and 0.1% (*v/v*) Triton X-100. For nuclear staining sections were incubated with To-PRO^®^-3 IODID (1:5000 in PBS for 10 min; Invitrogen, USA).

### MICROSCOPY

Traced entorhino-hippocampal fibers, *post hoc* identified recorded neurons, TNFα *in situ* hybridizations, and TNFα immunostainings were visualized using a Nikon Eclipse C1si laser-scanning microscope equipped with a 40× oil-immersion (NA 1.3, Nikon) and 60× oil-immersion (NA 1.4, Nikon) objective lens.

### QUANTIFICATION AND STATISTICS

Electrophysiological data were analyzed using pClamp 10.2 (Axon Instruments, USA) and MiniAnalysis (Synaptosoft, USA) software. All events were visually inspected and detected by an investigator blind to experimental condition. 250–350 events were analyzed per recorded neuron. No significant differences between age- and time-matched non-denervated cultures were observed (c.f., Vlachos et al., 2012a). Similarly no differences between 1 vs. 2 dpl, 3 vs. 4 dpl, TNFα treatment for 1 vs. 2 days and sTNFR treatments for 1 vs. 2 days or 3 vs. 4 days were observed. Therefore these data were pooled (control, 1–2 dpl, 3–4 dpl, 1–2 days or 3–4 days of treatment).

Quantitative PCR data were analyzed as described by Pfaffl (2001). GAPDH served as reference gene in this analysis. The qPCR assay efficiency was calculated with the StepOnePlus software (Applied Biosystems, USA) based on a dilution series of five samples for each assay. Data of age- and time-matched non-denervated control cultures were pooled.

TNFα and GFAP immunostainings were analyzed using the ImageJ software package^1^. Colocalization of TNFα and GFAP staining were assessed using the colocalization plugin for ImageJ^2^. GFAP, TNFα and colocalized pixel-areas were determined in defined regions of interest (ROIs, 40 × 40 μm) and expressed as percent of ROI area. ROIs were positioned in the GCL (detected by ToPRO nuclear staining) at 0–50 μm from GCL (=IML) or at a distance of 50–150 μm from the GCL (=OML; c.f. Vlachos et al., 2013b). Three ROIs were analyzed per layer in each culture and averaged values per culture were used for statistical comparison. No significant difference was observed between age- and time-matched non-denervated control cultures in these experiments.

Statistical comparisons were made using Mann–Whitney-test or Kruskal–Wallis-test followed by Dunn’s *post hoc* analysis. *P*-values of less than 0.05 were considered a significant difference. All values are expressed as mean ± standard error of the mean (SEM). In the figures, ^*^p < 0.05, ^**^p < 0.01, and ^***^p < 0.001; not significant differences are indicated with n.s.

### DIGITAL ILLUSTRATIONS

Confocal image stacks were exported as 2D-projections and stored as TIF files. Figures were prepared using Photoshop graphics software (Adobe, USA) and Inkscape^3^ (Free Software Foundation, USA). Image brightness and contrast were adjusted.

## RESULTS

### RECOMBINANT sTNFR IMPAIRS DENERVATION-INDUCED SYNAPTIC STRENGTHENING AT 3–4 dpl

To test for the role of TNFα in denervation-induced homeostatic synaptic plasticity a pharmacological approach was used first (**Figure 1**). Denervated and non-denervated wildtype cultures were treated immediately after the lesion with a recombinant sTNFR (10 μg/ml), which scavenges TNFα (Beattie et al., 2002; Stellwagen et al., 2005; Stellwagen and Malenka, 2006; Steinmetz and Turrigiano, 2010; Santello et al., 2011). mEPSCs were recorded from dentate granule cells at 1–2 and 3–4 dpl. During this time denervation-induced homeostatic synaptic plasticity is induced and synaptic strength increases (1–2 dpl) before it reaches a plateau (3–4 dpl; Vlachos et al., 2012a). Similar to untreated denervated cultures, a significant increase in mEPSC amplitudes was observed in sTNFR-treated wildtype cultures at 1–2 dpl (**Figures 1C–F**). However, at 3–4 dpl mEPSC amplitudes were not significantly increased in the sTNFR-treated group, while they remained high in untreated denervated cultures (mEPSC frequencies; untreated groups, control: 1.3 ± 0.2 Hz; 1–2 dpl: 2.1 ± 0.2 Hz, n.s.; 3–4 dpl: 2.6 ± 0.3 Hz, n.s.; sTNFR treated groups, control: 1.2 ± 0.2 Hz; 1–2 dpl: 2.4 ± 0.5 Hz, *p* < 0.05; 3–4 dpl: 1.2 Hz ± 0.2 Hz, n.s.; mEPSC rise and decay times not significantly changed after entorhinal denervation *in vitro*). These results indicated that TNFα could play an important role during the late/plateau phase of denervation-induced homeostatic synaptic strengthening.

### DENERVATION-INDUCED SYNAPTIC STRENGTHENING IS IMPAIRED AT 3–4 dpl BUT INTACT AT 1–2 dpl IN TNFα-DEFICIENT SLICE CULTURES

To confirm and extend these findings slice cultures prepared from TNFα-deficient mice (Pasparakis et al., 1996; Golan et al., 2004) were used. Prior to deafferentation we verified that the DG is innervated by entorhinal fibers in these cultures by anterograde tracing of the perforant path with Mini-Ruby (**Figure 2A**). Furthermore, by electrically stimulating the EC and simultaneously recording evoked EPSCs from dentate granule cells, we confirmed that entorhinal fibers form functional synapses with granule cells in these preparations (**Figure 2A**).

We then performed entorhinal denervation experiments and assessed changes in excitatory synaptic strength of dentate granule cells (**Figures 2C–E**). Similar to our pharmacological experiments a significant increase in mEPSC amplitudes was observed at 1–2 dpl in TNFα-deficient preparations. However, at 3–4 dpl mEPSC amplitudes returned back to baseline in these cultures (**Figures 2D,E**; mEPSC frequencies; control: 3.2 ± 0.4 Hz; 1–2 dpl: 2.2 ± 0.2 Hz, n.s.; 3–4 dpl: 4.1 ± 0.5 Hz, n.s.; mEPSC rise and decay times not significantly different compared to age- and time-matched wildtype littermates). Hence, TNFα could play an important role in maintaining rather than inducing a homeostatic increase in excitatory synaptic strength after denervation.

### TNFα-TREATMENT RESCUES THE ABILITY OF DENERVATED DENTATE GRANULE CELLS TO MAINTAIN INCREASED EXCITATORY SYNAPTIC STRENGTH AT 3–4 dpl IN TNFα-DEFICIENT SLICE CULTURES

**FIGURE 3:**
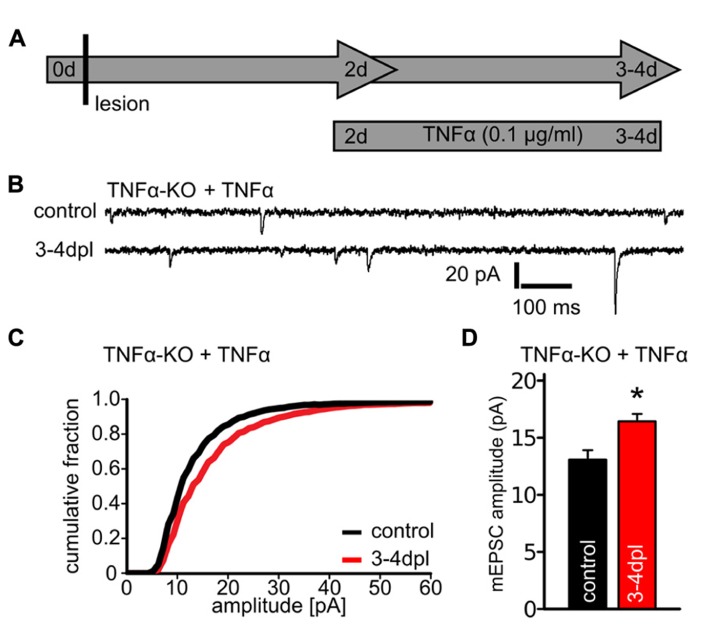
**Tumor necrosis factor alpha (TNFα)-treatment rescues the ability of denervated dentate granule cells to maintain increased excitatory synaptic strength at 3–4 dpl in TNFα-deficient slice cultures.** **(A)** Schematic illustrating the experimental design. Denervated TNFα-deficient slice cultures were treated at 2 dpl with TNFα (0.1 μg/ml) for 1 or 2 days and miniature excitatory postsynaptic currents (mEPSCs) were recorded (3–4 dpl). Age- and time-matched control cultures were not denervated but treated with the same concentration of TNFα for 1 or 2 days. **(B)** Sample traces of mEPSC recordings from granule cells of non-denervated (control) and denervated TNFα-deficient slice cultures which were treated with TNFα. **(C, D)** mEPSC amplitudes were increased in denervated TNFα-treated cultures (*n* = 17 neurons; five cultures), while the same treatment had no apparent effect on mEPSC amplitudes in non-denervated TNFα-deficient cultures (non-denervated TNFα-treated: 13.0 ± 0.7 pA; *n* = 8 cells; non-denervated untreated: 12.3 ± 0.8 pA; *n* = 12 cells; n.s.; from three to five cultures). Results of TNFα-treated wildtype cultures are reported in the text. Data represent mean ± SEM; *p < 0.05.

To test whether the impaired synaptic response in TNFα-deficient preparations at 3–4 dpl is indeed due to the lack of TNFα, another set of cultures was treated at 2 dpl with TNFα (0.1 μg/ml) for 1 or 2 days and mEPSCs were recorded (**Figure 3A**). In these experiments a persisting increase in mEPSC amplitudes was detected after denervation (**Figures 3B–D**), thus demonstrating that exogenous TNFα-treatment restores the ability of dentate granule cells in TNFα-deficient preparations to maintain increased excitatory synaptic strength following entorhinal denervation *in vitro*.

The same TNFα-treatment had no significant effect on mEPSC amplitudes in non-denervated. TNFα-deficient slice cultures (non-denervated untreated: 12.3 ± 0.8 pA; *n* = 12 cells; non-denervated TNFα-treated: 13.0 ± 0.7 pA; *n* = 8 cells; n.s.) and wildtype cultures (non-denervated untreated: 10.4 ± 0.3 pA; *n* = 10 cells; non-denervated TNFα-treated: 11.2 ± 0.5 pA; *n* = 9 cells; n.s.). Also, no significant increase in mEPSC amplitudes was observed when denervated wildtype cultures were treated with TNFα (denervated untreated: 16.2 ± 0.9 pA; *n* = 10 cells; denervated TNFα-treated: 18.6 ± 1.0 pA; *n* = 17 cells; n.s.). We concluded from these observations that TNFα (applied at a concentration of 0.1 μg/ml for 1 or 2 days to the culture medium) is not sufficient to induce a strengthening of excitatory postsynapses in dentate granule cells. Rather, TNFα seems to play a role in maintaining the increased excitatory synaptic strength, which is induced by entorhinal denervation *in vitro*.

### ENTORHINAL DENERVATION *IN VITRO* IS ACCOMPANIED BY CHANGES IN TNFα-mRNA LEVELS IN THE DENTATE GYRUS

The requirement of TNFα at 3–4 dpl but not 1–2 dpl indicated that denervation-induced synaptic plasticity is composed of molecularly distinct (or partially overlapping) phases. To test whether changes in mRNA expression reflect different phases after denervation, and to provide further evidence for the role of TNFα in denervation-induced synaptic plasticity with another technique, relative changes in TNFα-mRNA levels were assessed in the DG of denervated and age-matched non-denervated wildtype cultures. Of note, our previous work had revealed that a compensatory increase in excitatory synaptic strength is predominantly seen in the denervated OML at 3–4 dpl (Vlachos et al., 2012a). Thus, we predicted that TNFα-mRNA levels might also be increased in the deafferented zone.

**FIGURE 4:**
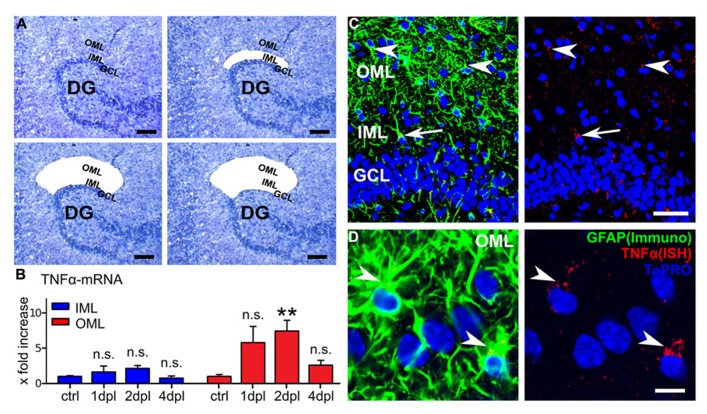
**Laser capture microdissection combined with qPCR reveals changes in TNFα-mRNA levels following entorhinal denervation *in vitro*.** **(A)** Laser capture microdissection (LMD) was employed to collect tissue from the inner molecular layer (IML; top right side), the outer molecular layer (OML; bottom left side), and the granule cell layer (GCL; bottom right side) of denervated cultures (at 1, 2, and 4 dpl) and non-denervated control cultures. **(B)** TNFα-mRNA levels were assessed in the isolated tissue using qPCR. An increase in TNFα-mRNA was observed in the OML at 2 dpl. TNFα-mRNA levels in the IML were not significantly changed. In the GCL TNFα-mRNA levels were below the detection threshold and no quantitative analysis of this layer was performed (*n* = 4–7 cultures per group). Data represent mean ± SEM; **p < 0.01; n.s., not significant. **(C)** *In situ* hybridization for TNFα-mRNA (red) and GFAP-immunostaining (green) at 2 dpl. TNFα-mRNA granules colocalized with GFAP-immunofluorescence. While most TNFα-mRNA/GFAP-positive cells were detected in the OML (arrowheads) and some in the IML (arrow), only a weak TNFα-mRNA signal was present in the GCL (mostly in colocalization with GFAP). These results indicate that astrocytes could be a major source of TNFα following entorhinal denervation *in vitro*. Scale bar: 50 μm. **(D)** Higher magnification of the OML. TNFα-mRNA granules can be identified in the soma and proximal processes of GFAP-positive cells (ToPRO nuclear staining, blue; *n* = 4 cultures). Scale bar: 10 μm.

To address this issue LMD was used to harvest tissue from the OML, the IML and the GCL at 1, 2, and 4 dpl (**Figure 4A**). The probes were obtained from the suprapyramidal blade of the DG, i.e., the region in which we have performed our experiments. Indeed, qPCR analysis revealed an increase in TNFα-mRNA levels in the tissue isolated from the denervated OML at 1 dpl which reached the level of significance at 2 dpl and returned back to baseline at 4 dpl, while in the IML no significant change was observed (**Figure 4B**). In the GCL TNFα-mRNA was below the detection threshold and for this reason no quantitative analysis of this layer could be performed. These results were consistent with our earlier findings on a layer-specific homeostatic synaptic response of dentate granule cells (Vlachos et al., 2012a) and indicated that changes in TNFα-mRNA levels (at 1–2 dpl) may precede the TNFα-dependent phase of denervation-induced synaptic plasticity.

### TNFα-mRNA IS DETECTED IN ASTROCYTES FOLLOWING ENTORHINAL DENERVATION *IN VITRO*

Previous work has demonstrated that astrocytes are activated following entorhinal denervation in the denervated zone (Rose et al., 1976; Steward et al., 1990, 1993; Fagan et al., 1997; Haas et al., 1999; Deller et al., 2000; Liu et al., 2005; Schäfer et al., 2005). Since pharmacological blockade of network-activity leads to an increase in glial TNFα (Stellwagen and Malenka, 2006) we tested for the possibility that astrocytes could be a source of TNFα in our experiments.

A different set of cultures was fixed at 2 dpl and *in situ* hybridizations for TNFα-mRNA were carried out. The slices were counterstained for GFAP, which served as a marker for astrocytes in these experiments (**Figures 4C,D**). Indeed, TNFα-mRNA was found predominantly colocalized with GFAP (**Figures 4C,D**). TNFα-mRNA granules were mostly found in somatic and perisomatic compartments, i.e., in proximal astrocytic processes (**Figure 4D**). Some TNFα-mRNA containing GFAP-positive cells and processes were also identified in the IML and occasionally in the GCL. The majority of GFAP and TNFα-mRNA positive cells, however, were detected in the denervated OML, consistent with our LMD-qPCR data (c.f., **Figure 4B**). Although these results do not rule out the possibility of neuronal or microglial TNFα, they indicated that astrocytes are a source of TNFα in our experimental setting.

### ASTROCYTIC TNFα IS INCREASED IN THE DENERVATED ZONE AT 4 dpl

**FIGURE 5:**
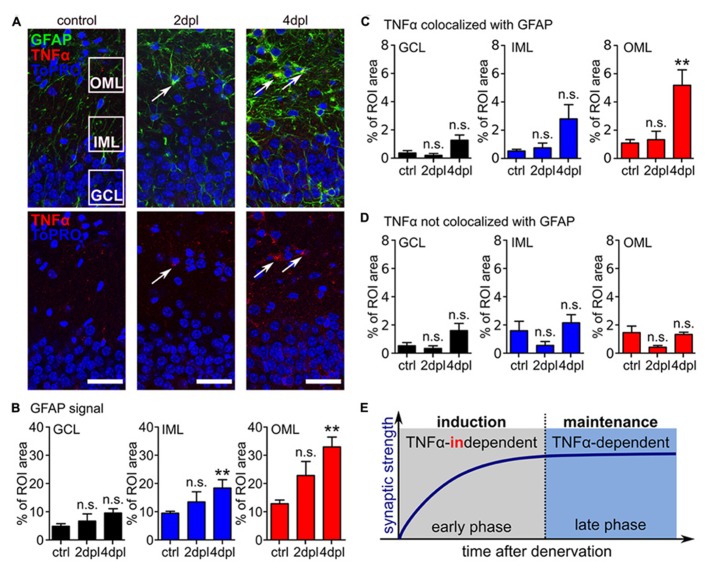
**Astrocytes are a source of TNFα following entorhinal denervation *in vitro*.** **(A)** Immunostainings for TNFα (red) and GFAP (green) reveal that both markers are upregulated post lesion compared to control. Arrows are pointing at examples of TNFα and GFAP-positive cells. The regions of interest (ROIs) used for analysis of changes in the outer molecular layer (OML), inner molecular layer (IML), and granule cell layer (GCL) are indicated (ToPRO nuclear staining, blue). Scale bars: 40 μm. **(B–D)** Quantification of immunofluorescence for GFAP, TNFα colocalized with GFAP and TNFα without GFAP in the GCL, IML, and OML. GFAP is upregulated predominantly in the OML following denervation **(B)**. Only the TNFα signal, which was colocalized with GFAP was significantly increased in the OML at 4 dpl **(C, D)**, suggesting that GFAP-positive astrocytes are a major source of TNFα following entorhinal denervation *in vitro* (*n* = 6–8 cultures per group). Data represent mean ± SEM; **p < 0.01; ***p < 0.001; n.s., not significant. **(E)** Entorhinal denervation *in vitro* reveals that denervation-induced homeostatic synaptic plasticity can be divided into an early synaptic strengthening response, which does not require TNFα, and a late TNFα-dependent synaptic strengthening response. TNFα plays an important role in maintaining the increased excitatory synaptic strength during the late-phase.

In light of these results we tested whether TNFα protein levels increase after entorhinal denervation *in vitro*. Cultures fixed at 2 and 4 dpl were immunostained for TNFα as well as GFAP and changes in immunofluorescence were determined. Consistent with the results obtained from *in vivo* entorhinal denervation experiments (Steward et al., 1990, 1993; Burbach et al., 2004) a significant increase in GFAP signal was observed after entorhinal denervation *in vitro* (compared to non-denervated age- and time-matched control cultures; **Figures 5A,B**). We then evaluated changes in TNFα immunofluorescence colocalized with GFAP (**Figure 5C**) and *not* colocalized with GFAP fluorescence (**Figure 5D**). Indeed, only the TNFα signal which colocalized with GFAP was significantly increased in the OML at 4 dpl. Together, these data suggested that activated astrocytes are a major source of TNFα following entorhinal denervation *in vitro*.

## DISCUSSION

The results of the present study demonstrate that TNFα is required for the maintenance (3–4 dpl) but not the induction (1–2 dpl) of homeostatic synaptic plasticity following entorhinal denervation *in vitro*. This finding indicates that the postlesional reorganizationof denervated neuronal networks might be composed of molecularly distinct (or partially overlapping) phases. TNFα appears to play an important role during the late/plateau phase of denervation-induced homeostatic synaptic plasticity (**Figure 5E**).

### THE ROLE OF TNFα IN DENERVATION-INDUCED HOMEOSTATIC SYNAPTIC PLASTICITY

TNFα has been implicated as one of the secreted factors, which regulate pharmacologically induced homeostatic synaptic plasticity (Stellwagen and Malenka, 2006). Evidence has been provided that prolonged TTX-treatment leads to TNFα release from glia cells, which in turn induces an increase in excitatory synaptic strength (Stellwagen and Malenka, 2006) and also regulates inhibitory synaptic strength (Stellwagen et al., 2005; Pribiag and Stellwagen, 2013). The work by Steinmetz and Turrigiano (2010) indicated that TNFα could have permissive rather than instructive effects in TTX-induced synaptic scaling. In this earlier study (Steinmetz and Turrigiano, 2010) compelling experimental evidence was provided using pharmacological approaches that TNFα is necessary during prolonged (>24 h) but not early (~6 h) activity blockade in dissociated cortical neurons. This observation is comparable with the results of the present study, in which homeostatic synaptic strengthening was induced by entorhinal denervation in slice cultures prepared from TNFα-deficient mice. While a compensatory increase in mEPSC amplitudes was observed at 1–2 dpl, increased excitatory synaptic strength was not seen at 3–4 dpl in granule cells of TNFα-deficient cultures. Moreover, bath-application of TNFα (0.1 μg/ml) rescued in these cultures the ability to maintain increased synaptic strength at 3–4 dpl, while having no discernible effect on mEPSC amplitudes in non-denervated control cultures. Thus, TNFα can be considered an important regulatory molecule, which controls the ability of denervated neurons to maintain a homeostatic increase in excitatory synaptic strength. It will now be important to identify the downstream signaling pathways through which TNFα controls this ability of neurons (for a recent review on metaplasticity see Hulme et al., 2012) and to compare these findings with data obtained in other testing conditions (c.f., review by Santello and Volterra, 2012).

### ASTROCYTES ARE A SOURCE OF TNFα IN THE DENERVATED ZONE

In our earlier work we reported evidence that excitatory synaptic strength is predominantly increased in the denervated layer at 3–4 dpl (Vlachos et al., 2012a). This finding is in line with the layer-specific increase in TNFα-mRNA levels in the OML at 2 dpl, as revealed by a combination of LMD and qPCR. Using *in situ* hybridization the TNFα-mRNA signal could be localized to GFAP-positive cells, i.e., activated astrocytes. Immunostaining for TNFα and GFAP further corroborated these findings and revealed an enrichment of TNFα in GFAP-positive astrocytes in the denervated zone at 4 dpl. Taken together, we conclude that astrocytes are a major source of TNFα in our experimental setting, although this finding does not rule out a contribution from other TNFα sources, e.g., microglia (Lambertsen et al., 2001; Fenger et al., 2006) or neurons (Harry et al., 2008; Janelsins et al., 2008) *in vivo*.

Astrocytes activated by entorhinal denervation delineate the denervated OML of the DG (Fagan et al., 1997; Haas et al., 1999; Deller et al., 2000; Liu et al., 2005; Schäfer et al., 2005). They are thus in an ideal spatial position to regulate the remodeling occurring within the denervated layer. Based on the findings of this study, we propose that activated astrocytes could play an important role in the regulation of functional changes after entorhinal denervation. Their presence in the OML could lead to a longer lasting homeostatic increase in the strength of surviving excitatory synapses, which could uphold the level of excitatory drive to a denervated granule cell, e.g., until this cell is reinnervated by sprouting excitatory axons. Whether astrocyte-derived TNFα is also required in other testing conditions in which local homeostatic responses have been reported (Sutton et al., 2006; Branco et al., 2008; Hou et al., 2008; Kim and Tsien, 2008; Deeg and Aizenman, 2011; Mitra et al., 2012) remains unknown at present and may depend on the particular experimental approach, which is used to induce homeostatic plasticity.

### DENERVATION-INDUCED HOMEOSTATIC SYNAPTIC PLASTICITY *IN VITRO* IS COMPOSED OF MOLECULARLY DISTINCT PHASES

A key finding of our study is the observation that granule cells of TNFα-deficient preparations are not impaired in their ability to express homeostatic synaptic plasticity at 1–2 dpl, which is supported by our pharmacological experiments in wildtype cultures in which we have used sTNFR to scavenge TNFα. This observation indicates that distinct signaling pathways could control different phases of denervation-induced homeostatic synaptic plasticity (induction vs. plateau vs. down-scaling; c.f., Vlachos et al., 2012a). Apparently, TNFα-signaling is required during the plateau-phase of denervation-induced homeostatic synaptic plasticity, i.e., at 3–4 dpl. Although the signals which orchestrate different phases of homeostatic synaptic plasticity remain unknown, our LMD-qPCR data suggest that regulatory pathways may act, at least in part, at the level of gene expression. Hence, a systematic comparison of the differences in mRNA levels (and protein expression at the same or later time points; Dieterich et al., 2006; Cajigas et al., 2012) between different layers at different points in time after entorhinal denervation (and/or pharmacological treatments) may allow to identify novel candidate regulatory molecules involved in local and global homeostatic synaptic plasticity.

### THE ROLE OF TNFα-DEPENDENT HOMEOSTATIC SYNAPTIC PLASTICITY IN NEUROLOGICAL DISEASES

Since entorhinal denervation was employed in our study to induce homeostatic synaptic strengthening it is conceivable that TNFα-mediated homeostatic synaptic plasticity could be of relevance for a broad range of neurological diseases, which are accompanied by a deafferentation of neurons. In fact, several neurological diseases are associated with increased TNFα-expression levels (Sriram and O’Callaghan, 2007). While it has been proposed that the pathological release of TNFα could lead to alterations in physiological network functions (Small, 2008; Park et al., 2010; Montgomery and Bowers, 2012), the biological consequences of TNFα-mediated homeostatic synaptic plasticity remain unclear. On the one hand the maintenance of homeostatic synaptic plasticity could be beneficial by promoting stability during the post-lesional reorganization of denervated networks. On the other hand a persisting increase in excitatory synaptic strength could also aggravate the susceptibility for runaway excitation and epileptic discharges in lesioned neuronal networks (Savin et al., 2009). It therefore remains to be shown whether targeting TNFα-signaling could have beneficial or detrimental effects for the course of a neurological disease (Tobinick et al., 2006; McCoy and Tansey, 2008; Zhao et al., 2011).

### THE ROLE OF TNFα IN DENERVATION-INDUCED STRUCTURAL REMODELING

In one of our recent studies (Vlachos et al., 2013a) we were able to provide experimental evidence which suggests that denervation-induced homeostatic synaptic plasticity can lead to heterosynaptic competition between strengthened excitatory synapses and newly formed dendritic spines, i.e., spines formed after denervation. This mechanism seems to delay spine density recovery after denervation via the destabilization of new spines (Vlachos et al., 2013a). Noteworthy, spine destabilization was observed in the denervated zone (Vlachos et al., 2012b, 2013a) as was also the case for homeostatic synaptic strengthening at 3–4 dpl (Vlachos et al., 2012a) and the increase of TNFα-mRNA levels and TNFα-immunofluorescence signal (this study). Hence, it will now be interesting to study the effects of TNFα on spine numbers and dynamics following entorhinal denervation *in vitro* and to assess whether inhibition of TNFα-signaling accelerates spine density recovery after denervation.

## Conflict of Interest Statement

The authors declare that the research was conducted in the absence of any commercial or financial relationships that could be construed as a potential conflict of interest.

## Footnotes

1

2

3
