# Corrigendum: Tumor necrosis factor alpha maintains denervation-induced homeostatic synaptic plasticity of mouse dentate granule cells

**DOI:** 10.3389/fncel.2014.00230

**Published:** 2014-08-12

**Authors:** Denise Becker, Nadine Zahn, Thomas Deller, Andreas Vlachos

**Affiliations:** Institute of Clinical Neuroanatomy, Neuroscience Center, Goethe-University FrankfurtFrankfurt, Germany

**Keywords:** entorhinal cortex lesion, homeostatic synaptic scaling, astrocytes, brain injury, organotypic slice culture

We noticed that in **Figure 2C** of our article the sample traces shown for non-denervated controls and for denervated TNFα-deficient preparations at 3–4 days post lesion (dpl) are identical. Upon re-examination of the original recordings, we found that this sample trace was taken from a denervated dentate granule cell (“3–4 dpl group”). The corrected figure showing a sample trace of a non-denervated control is now presented. We apologize for the mistake and for any inconvenience caused to the readers.

**Figure 1:**
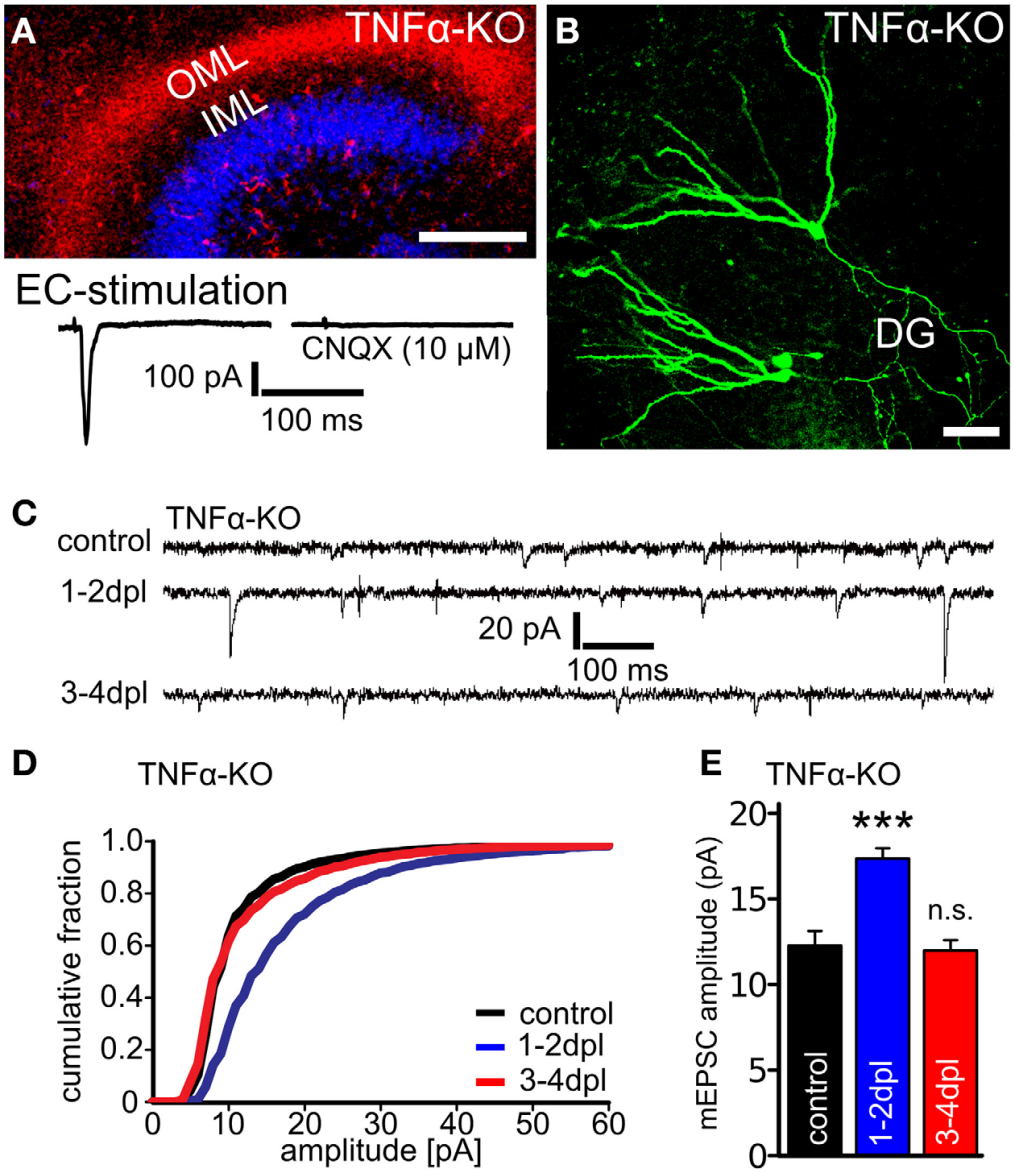
**Figure legend same as in the original article: Denervation-induced homeostatic synaptic strengthening is not observed in granule cells of TNFα-deficient slice cultures at 3–4 dpl. (A)** Mini-Ruby tracing of entorhino-hippocampal axons (red; ToPRO nuclear staining, blue) and electrical stimulations of the entorhinal cortex (EC) while recording evoked EPSCs from dentate granule cells revealed an intact and functional entorhino-hippocampal projection in slice cultures prepared from TNFα-deficient mice (TNFα-KO; three independent experiments each, up to 50 traces averaged per neuron). Evoked EPSCs (amplitude: 369 ± 102 pA) could be blocked by the AMPA-receptor antagonist CNQX (10 μM; amplitude: 9.6 ± 2.1 pA). Scale bar: 200 μm. **(B)** Patched granule cells were filled with biocytin and *post hoc* identified using Alexa568- or Alexa488-streptavidin. Scale bar: 50 μm. **(C–E)** Whole-cell patch-clamp recordings from granule cells of TNFα-deficient slice cultures revealed an increase in the mEPSC amplitudes at 1–2 dpl but not at 3–4 dpl (*n* = 12–16 neurons per group, from six to eight cultures each). Data represent mean ± s.e.m.; ^***^p < 0.001; n.s., not significant.

## Conflict of interest statement

The authors declare that the research was conducted in the absence of any commercial or financial relationships that could be construed as a potential conflict of interest.

